# Antiviral activity of green synthesized selenium nanoparticles alone and in combination with chitosan against SARS-CoV-2

**DOI:** 10.1186/s11671-025-04420-6

**Published:** 2026-01-19

**Authors:** Mohamed M. El-Zahed, Sarah A. Kandel, Mahmoud E. Khalifa

**Affiliations:** Botany and Microbiology Department, Faculty of Science, Damietta University, Damietta, 34517 Egypt

**Keywords:** Antiviral, COVID-19, SARS-CoV-2, Selenium, Nanoparticles, Chitosan, Nanocomposite

## Abstract

Nanobiotechnology is increasingly used to control viral diseases such as COVID-19, with selenium nanoparticles (SeNPs) and their composite with chitosan (CS) gaining attention for their broad bioactivity and potential as antiviral agents, but challenges related to synthesis methods, cytotoxicity, and mechanistic understanding remain. In this study, we report a novel, green biosynthesis of SeNPs using *Limosilactobacillus fermentum*, followed by functionalization with chitosan to produce Se/CS nanocomposites with enhanced antiviral performance against SARS-CoV-2. *L. fermentum* was used to biosynthesize SeNPs, providing a rapid, safe, and environmentally friendly approach. The production process was optimized by testing different parameters such as concentrations of Na_2_SeO_3_, temperature, ratios between cell-free bacterial metabolites and Na_2_SeO_3_, and pH. UV–Vis spectroscopy, FT-IR, XRD, Zeta potential, and TEM studies confirmed the successful synthesis of Se/CS NC, with a distinctive peak at 266 nm. FT-IR also showed that proteins were present as capping and stabilizing agents in Se/CS NC. Se/CS NC has a high zeta potential with a negative net surface charge of − 21.84 ± 4.7 mV, giving Se/CS NC great stability. Se/CS NC had an average particle size of 38.19 nm and exhibited a crystalline morphology. Biological assays in SARS-CoV-2-infected Vero E6 cells revealed that SeNPs alone displayed dose-dependent cytotoxicity, reducing cell viability above 125 µg/ml. In contrast, Se/CS NC maintained over 96% cell viability at all tested concentrations and demonstrated potent antiviral activity, achieving over 95% inhibition of viral replication at concentrations ≥ 250 µg/ml. Studies identified virucidal action as the primary antiviral mechanism, with 47.4% inhibition at 500 µg/ml. To the best of our knowledge, this study provides the first experimental evidence that green-synthesized Se/CS NC produced by *L. fermentum* can effectively inhibit SARS-CoV-2, highlighting their potential as a safe, eco-friendly antiviral candidate for future COVID-19 therapies and pharmaceutical applications. This demonstrates a direct application of nanotechnology in combating COVID-19 by suppressing viral replication and maintaining host cell viability.

## Introduction

Novel antiviral medicines with great efficacy and safety have been sought due to the COVID-19 pandemic, caused by severe acute respiratory syndrome coronavirus 2 (SARS-CoV-2), especially since new strains of the virus are constantly appearing. SARS-CoV-2 infection damages cells and weakens the host’s immune response, which leads to systemic inflammation and multiple organ involvement [1]. New drugs that simultaneously stop viruses from replicating and modulate the body’s immune system are required to address these issues [2–4].

Among nanomaterials, selenium nanoparticles (SeNPs) are known for their antiviral, antioxidant, and immunomodulatory properties [5]. SeNPs are a popular non-metallic NP derived from Selenium (Se) molecules essential to human and animal cells. Se molecules in the Se^0^ oxidation state have low toxicity, high bioavailability, greater reactivity, and lower dosage compared to Se^6+^ and Se^4+^ [6]. SeNPs have demonstrated efficacy against various viruses, including H1N1 influenza and human immunodeficiency virus (HIV) [7, 8] and show promise in treating drug-induced toxicities, inflammatory disorders, liver fibrosis, diabetes, and cardiovascular disease [9]. However, their clinical application is limited by potential cytotoxicity at higher concentrations [10].

To address this limitation, surface modification of SeNPs with biocompatible polymers has been explored. Chitosan (CS), the only positively charged organic polymer, is biodegradable and biocompatible [11] and can inhibit viruses, yeasts, fungi, and bacteria [12]. New nanotechnology-based drug delivery technologies include NPs with therapeutic chemicals and adsorbed polymer matrices. Nanodrugs reduce adverse effects, increase bioavailability, and improve therapeutic pharmaceutical absorption [13]. CS offers antiviral, wound dressing, medication delivery, and tissue regeneration applications [14] and it can interact with viral particles and host cell membranes, thereby inhibiting viral entry and replication, and has been shown to potentiate the antiviral activity of metal nanoparticles while reducing their cytotoxicity [15].

Different approaches are used to synthesize NPs. Green synthesis for NPs is gaining popularity due to its low cost, simplicity, safety, non-toxicity, and high productivity [16], compared to conventional chemical and physical methods, which have drawbacks like long synthesis times, high production costs, purification issues, and toxic byproducts [17]. Biosynthesis of NPs employing bacteria, actinobacteria, algae, fungi, and yeasts has been the subject of recent research [18–20]. Probiotic lactic acid bacteria (LAB) are promising candidates for NP biosynthesis because they produce bioactive compounds, enzymes, and reducing agents that help form metallic NPs [21]. *Lactobacillus*, *Lactococcus*, and *Bifidobacterium* species are safe, biocompatible, and widely used in industry, making them excellent for green synthesis [22]. Different LAB strains have been reported to biosynthesize NPs from 50 to 100 nm (*Streptococcus thermophilus*), 100–200 nm (*Lactobacillus* sp.), and 400–500 nm (*Bifidobacterium* sp.) [23].

The *L. fermentum* is an extensively studied probiotic with a long history of safe human consumption, possessing a Generally Recognized as Safe (GRAS) status. This choice is critical because using a known safe organism minimizes regulatory hurdles and maximizes the biocompatibility of the final SeNPs product, which is a key advantage over using environmental or pathogenic microorganisms. The primary reason for its selection is its robust and unique metabolic reducing machinery. *Lactobacillus* species, including *L. fermentum*, are known to produce and secrete a complex cocktail of reducing agents into their cell-free supernatants, including sulfhydryl groups (cysteine-rich proteins), NAD(P)H-dependent enzymes, and organic acids. This diverse reducing potential is essential for efficiently driving the reduction of SeO_3_^2−^​ to Se^0^ NPs [24]. Furthermore, the extracellular polymeric substances (EPS) and secreted proteins in the *L. fermentum* cell-free supernatants act as natural capping and stabilizing agents. This negates the need for external synthetic stabilizers, resulting in highly stable, well-dispersed nanoparticles which is a major advantage over many other green synthesis methods [22]. The biosynthesis of nanoparticles by LAB is primarily mediated by secreted metabolites that act as reducing and stabilizing agents. The biological activity and physicochemical properties of the resulting nanoparticles are strongly influenced by the nature and concentration of these metabolites [25].

Despite this promising data, direct comparative studies of SeNPs and selenium/chitosan nanocomposite (Se/CS NC) against SARS-CoV-2 remain limited. In this study, green-synthesized SeNPs and Se/CS NC were tested for cytotoxicity, antiviral effectiveness, and mechanisms of action in SARS-CoV-2-infected Vero E6 cells. Comparing these two nanoparticles’ therapeutic potential was done to help develop safer and more effective nanotechnology-based COVID-19 therapies. Thus, a green synthesis approach for producing chitosan-functionalized SeNPs using probiotic LAB strains was developed. The method aims to combine the antiviral and antioxidant properties of selenium, the biocompatibility and functional benefits of chitosan, and the eco-friendly, sustainable synthesis capabilities of LAB, offering a novel and potentially safer strategy for producing bioactive nanomaterials with enhanced therapeutic potential.

## Materials and methods

### Materials

*Limosilactobacillus fermentum* (AC: OR553490) was kindly obtained from the Microbiology Lab, Faculty of Science, Damietta University. Sodium selenite (Na_2_SeO_3_, MW: 172.94, anhydrous) was purchased from VWR Prolabo (France). De Man–Rogosa–Sharpe (MRS) medium was purchased from Oxoid Ltd. (UK). Chitosan (MW 50–190 KDa, deacetylation degree: ≥85%) was purchased from Techno Pharmchem. (India). Dulbecco modified Eagle’s medium (DMEM) was obtained from Lonza (Basel, Switzerland).

### Extracellular biosynthesis of SeNPs

*L. fermentum* slants were sub-cultured on De Man–Rogosa–Sharpe (MRS) agar plates, then were inoculated in nutrient broth medium and incubated for 24 h at 37 °C at a rotation rate of 150 rpm. The 24 h grown bacterial suspensions were centrifuged at 4000 rpm for 20 min using a low-speed centrifuge to collect the culture supernatants. 1 mM of Na_2_SeO_3_ solution was prepared and added into the flasks of bacterial metabolite by the ratio 1:1 (v/v), then incubated in a shaking incubator at 150 rpm at 37 °C until the color changed from colorless to red color (Mohamed & El-Zahed, 2024). All produced SeNPs reaction mixtures were measured spectrophotometrically to detect the biosynthesis processes using a double-beam UV–Vis spectrophotometer V-760 (JASCO, UK). The nano-colloidal solution was centrifuged at 4000 rpm for 15 min to collect SeNPs. The obtained NPs were washed three times using distilled water and then dried using a freeze-dryer for further experiments.

### Optimization of SeNPs production

Various concentrations of Na_2_SeO_3_ (10–100 mM), a range of temperatures (10–60 °C), different ratios between cell-free bacterial metabolites and Na_2_SeO_3_ (1:1–1:16 v/v%), and different pH levels (5–9) were tested to determine the ideal conditions for the biosynthesis of Se NPs. Spectrophotometric measurements of the production rate and concentration of Se NPs were made to clarify the optimized parameters for the bio-formation of Se NPs.

### Preparation of Se/CS NC

Se/CS NC were green synthesized throughout the coating of SeNPs by CS. Initially, 60 mg of CS was dissolved in 1% (v/v) acetic acid under magnetic stirring for 1 h at room temperature to ensure complete dissolution. Subsequently, 60 mg of SeNPs were added to the CS solution, and the mixture was stirred overnight to facilitate uniform dispersion [26]. The resulting mixture was dried using a freeze-dryer until complete solvent evaporation, yielding the Se/CS NC powder.

### Characterization of SeNPs and Se/CS NC

Optimized SeNPs and Se/CS NC were characterized spectrophotometrically using a double beam spectrum UV–Vis spectrophotometer (V-760, JASCO, UK), Fourier transform infrared spectroscopy (FT-IR, JASCO, UK), and X-ray diffraction (XRD) analysis which was performed using an X-ray diffractometer (LabX XRD-6000, Shimadzu, Japan). Zeta potential was measured using a Malvern Zetasizer Nano-ZS90 (Malvern, UK), and transmission electron microscopy (TEM) was performed with a JEOL JEM-2100 microscope (Japan).

### MTT cytotoxicity assay

The cytotoxicity of the prepared nanomaterials was evaluated using the MTT assay to determine concentrations causing 50% cell death (CC₅₀). Our SeNPs and Se/CS NC were fully water-soluble and required no organic solvents. After dispersing nanomaterials in sterile distilled water, we prepared working dilutions in DMEM. Vero E6 cells (chosen for SARS-CoV-2 susceptibility) were seeded in 96-well plates (100 µl/well at a density of 3 × 10^5^ cells/ml) and incubated for 24 h at 37 °C with 5% CO₂. We added nanomaterial dilutions in triplicate and incubated for a further 24 h. After three PBS rinses, MTT (20 µl of 5 mg/ml) was applied to the monolayers for four hours. The formazan crystals were then dissolved in 200 µl acidified isopropanol. A multi-well plate reader measured formazan solution absorbance at 540 nm. The percentage of cytotoxicity compared to untreated cells was calculated using the following equation:

$$\:\%\:cytotoxicity=\:\frac{\left(absorbance\:of\:untreated\:cells\:-absorbance\:of\:treated\:cells\right)}{absorbance\:of\:untrated\:cells}\times\:100$$

### Antiviral activity (IC₅₀ determination)

Vero E6 cells (2.4 × 10^4^ cells/well) were incubated overnight at a humidified 37 °C incubator under 5%CO_2_ conditions for antiviral assessment. The cells were washed once with 1x PBS and infected with SARS-CoV-2 (hCoV-19/Egypt/NRC-03/2020, GISAID: EPI_ISL_430820) for one hour at room temperature. The cells were overlaid with 100 µl DMEM containing varying concentrations of the tested nanomaterials. Following 72-hour incubation, we fixed cells with 100 µl 4% paraformaldehyde for 20 min and stained plaques with 0.1% crystal violet for 15 min at RT. The crystal violet dye was then dissolved using 100 µl of absolute methanol per well, and the optical density of the color was measured at 570 nm. The IC₅₀ (concentration inhibiting 50% plaque formation) was calculated from dose-response curves.

### Mechanism of action studies

To gain insight into how the selenium-chitosan nanocomposite (Se/CS NC) interferes with the SARS-CoV-2 replication cycle, we conducted a series of targeted assays to distinguish between direct virucidal effects, inhibition of viral adsorption, and suppression of viral replication, enabling us to map the primary antiviral action of our nanocomposite.

#### Virucidal activity assay

The virucidal assay was designed to determine whether Se/CS NC could directly inactivate SARS-CoV-2 particles before encountering host cells, as previously described by Schuhmacher et al. [27]. For this, virus suspensions containing 10^3^ plaque-forming units (PFU) per well were mixed with Se/CS NC at selected non-cytotoxic concentrations (125, 250, and 500 µg/ml) and incubated at 37 °C for one hour. This pre-incubation allows the nanocomposite to interact with the viral envelope and proteins without host cells, potentially leading to structural disruption or loss of infectivity.

Following this treatment, the virus-nanocomposite mixtures were transferred onto confluent Vero E6 cell monolayers in 6-well plates and adsorbed for one hour at 37 °C. After adsorption, the inoculum was removed, and the cells were overlaid with DMEM/2% agarose. This limited virus propagation and made distinct plaques easier to form. Plates were incubated for 72 h to form plaques. Subsequently, the cells were fixed with 10% formaldehyde and stained with crystal violet to visualize plaques. The number of plaques in treated wells was compared to virus-only controls, and the percent reduction was calculated to quantify the virucidal effect.

#### Viral adsorption Inhibition assay

To determine whether Se/CS NC interferes with the ability of SARS-CoV-2 to attach to host cell surfaces, we performed a viral adsorption inhibition assay as described by Zhang et al. [28]. Vero E6 cells were seeded in 6-well plates at a 1 × 10⁵ cells/mL density and incubated overnight at 37 °C with 5% CO_2_. The following day, the cell monolayers were pre-chilled to 4 °C, which permits viral binding but prevents internalization, ensuring that only the adsorption step is assessed. Se/CS NC at various non-toxic concentrations was added to the cells along with 300 µl of infection medium and incubated at 4 °C for one hour. This step allows the nanocomposite to interact with the cell surface, potentially blocking viral receptors or modifying membrane properties. After washing the cells three times with cold PBS to remove unbound nanocomposite, hCoV-19/Egypt/NRC-03/2020 (10^3^ PFU/well) was added and allowed to adsorb for another hour at 4 °C. Unbound virus was removed by washing, and cells were overlaid with 3 ml 2× DMEM containing 2% agarose. Plates were incubated at 37 °C for plaque development. After fixation and staining, plaques were counted, and the reduction in plaque number compared to virus-only controls was used to assess the degree of adsorption inhibition.

#### Viral replication Inhibition assay

A post-entry replication inhibition assay was conducted to evaluate whether Se/CS NC can suppress SARS-CoV-2 replication after the virus has entered host cells, as described by Kuo et al. [29]. Vero E6 cells were seeded and grown to confluence in 6-well plates at 37 °C in 5% CO_2_ for 24 h. The cells were infected with hCoV-19/Egypt/NRC-03/2020 at a multiplicity of infection (MOI) yielding approximately 1000 PFU per well and incubated at 37 °C for one hour to allow viral entry.

After adsorption, the excess virus was removed by washing the cells three times with PBS. Se/CS NC at the indicated concentrations was then added to the infected cells in 300 µL of infection medium and incubated for one hour at 37 °C. This step ensures that the nanocomposite is present during the early stages of viral replication but only after viral entry. Following this, 3 mL of 2× DMEM containing 2% agarose was overlaid onto the cells, and plates were incubated at 37 °C for 72 h to allow plaque formation. As with the other assays, plaques were fixed, stained, and counted. The percent reduction in plaque number relative to infected controls indicated the extent of replication inhibition.

## Results and discussion

### Biosynthesis of SeNPs

**Fig. 1:**
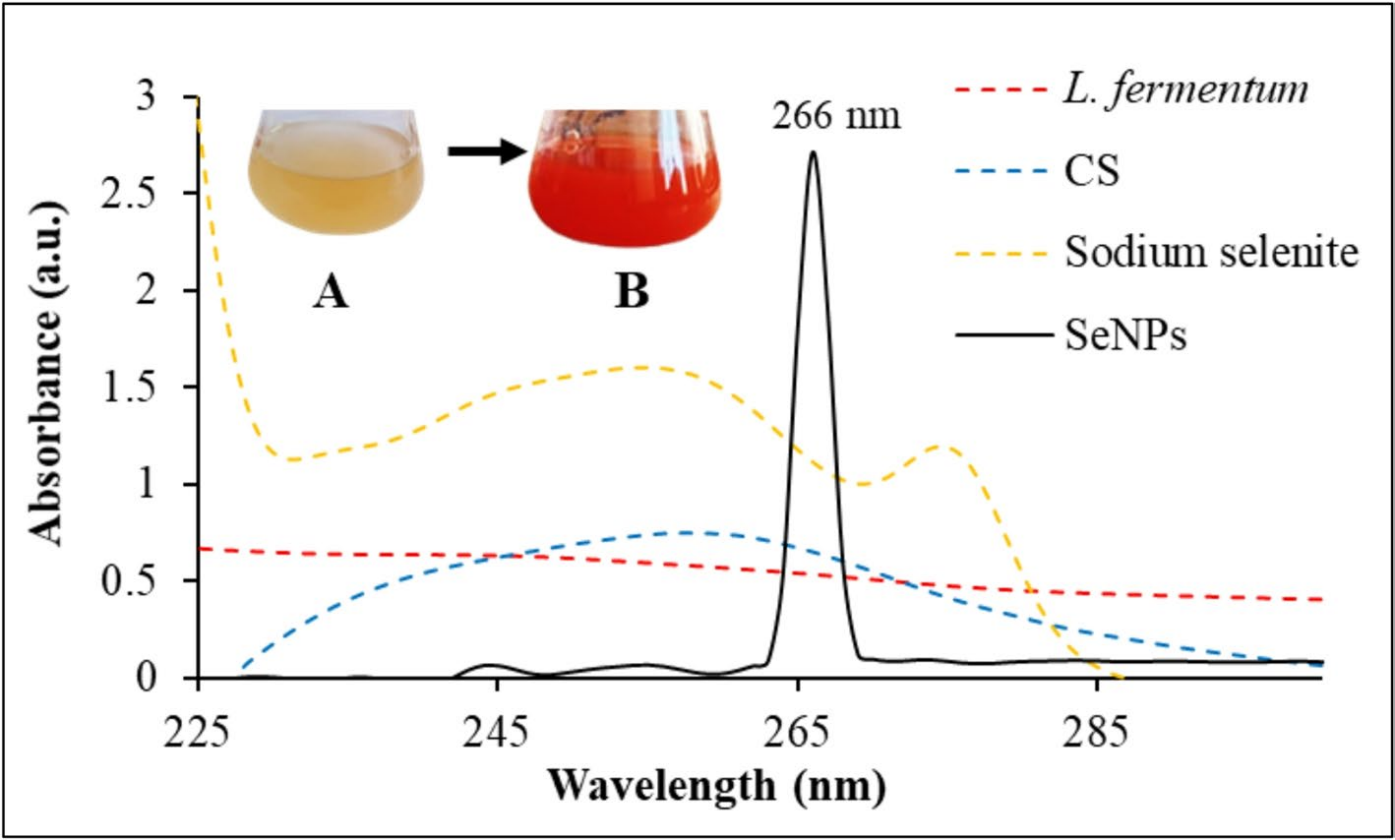
UV–Vis spectroscopy and color change observation during the extracellular biosynthesis of SeNPs using *L. fermentum*. The color changed from pale yellow (**A)** to red color (**B)** after the incubation time indicating the successful formation of SeNPs

Bacteria can biosynthesize various NPs through a variety of processes, according to numerous mechanisms [30]. Using NADH-dependent enzymes to bio-reduce metal ions into nanometals is one of the reported pathways for NPs production under dark conditions [31]. The biosynthetic process may occur either extracellularly or intracellularly. Nevertheless, the extracellular methods are preferred because the intracellular methods necessitate more steps, including cell disintegration, extraction, and purification of the synthesized NPs [32]. The current study reported that SeNPs were extracellularly biosynthesized using *L. fermentum* within 3 days at 37 °C and under dark conditions. The first indication of SeNPs formation was the medium’s color changing from pale yellow to red after the incubation time. The appearance of a distinctive red is due to the excitation of surface plasmon vibrations known as surface plasmon resonance (SPR) of SeNPs [33]. Nevertheless, the exact process by which bacteria generate SeNPs is still unknown. Previous studies have shown that the formation of metal NPs depends critically on NADH and the NADH-dependent nitrate reductase enzyme [34]. Additionally, reductases can serve as capping agents, ensuring the formation of nanostructures that are thermodynamically stable [35]. *L. fermentum* has been reported to produce NADH and NADH-dependent reductase enzymes [36, 37]. The UV-visible spectrometer absorption peak was analyzed by setting the wavelength range between 200 and 500 nm, and the peak appeared at 266 nm, which indicates the SPR of SeNPs (Fig. 1). This absorption peak is consistent with the SeNPs absorption band reported by Greeshma and Mahesh [38] who observed an absorbance peak of 266.5 nm, and Srivastava and Mukhopadhyay [39] who observed an absorbance peak at 270 nm. While other studies reported the absorption peaks of SeNPs at 350 nm [40] and 360 nm [41].

### Optimization of SeNPs

**Fig. 2:**
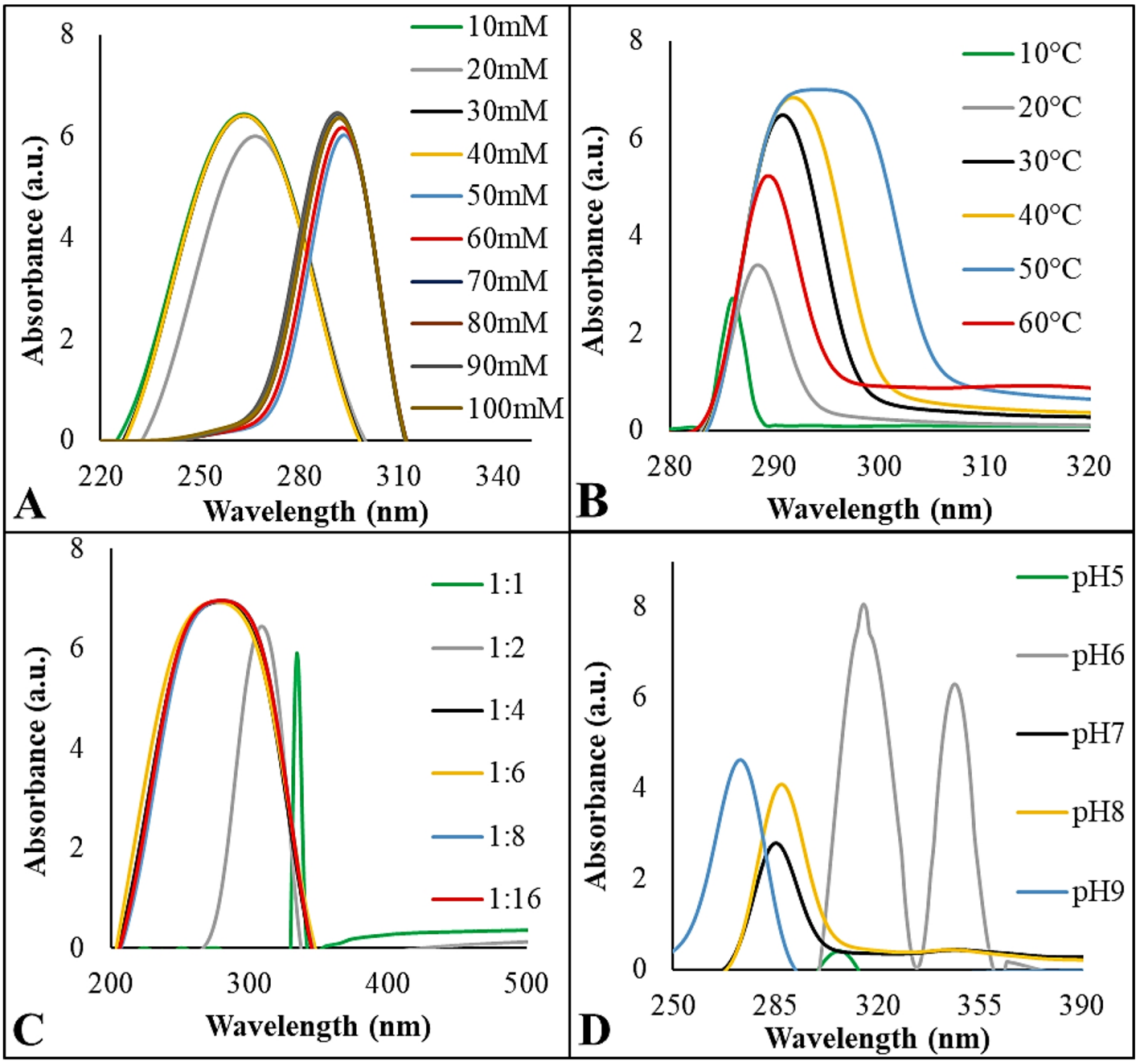
Optimization of the biosynthesized SeNPs production using *L. fermentum* by testing different concentrations of Na_2_SeO_3_ (**A)** temperatures, (**B)** mixing ratio between *L. fermentum* cell-free supernatant and Na_2_SeO_3_ (**C**), and pH of the reaction mixture (**D**)

To identify the optimum conditions for SeNPs production, several factors, including concentration of Na_2_SeO_3_ (10–100 mM), pH levels, temperatures (10–60 °C), and mixing ratios between bacterial supernatant and Na2SeO3, were tested (Fig. 2). The absorbance increased by increasing the concentration of Na_2_SeO_3_, reaching a sharp and intense SPR peak at 30 mM, indicating optimal SeNPs formation (Fig. 2A). Higher concentrations led to broader peaks and a red shift, suggesting particle aggregation. Therefore, 30 mM was selected as the optimal concentration, which is consistent with a previous study that reported optimal SeNPs formation at a similar concentration [42]. The absorbance intensity increased by raising the temperature levels in the range of 30–40 °C, showing a sharp and distinct peak, indicating efficient NPs formation (Fig. 2B). However, temperatures above 40 °C resulted in broadened peaks and red shift due to protein denaturation and particle aggregation. This suggests that higher temperatures have negatively affected SeNPs’ stability and synthesis efficiency. Thus, 30–40 °C was selected as the optimal temperature range, matched with Puri et al. [43]. The mixing ratio between *L. fermentum* cell-free supernatant and Na_2_SeO_3_ also significantly influences the biosynthesis efficiency and SeNPs quality (Fig. 2C). In the current study, the 1:1 volume ratio resulted in a distinct red coloration and a sharp peak, indicative of well-formed and stable SeNPs. This suggests optimal metabolic activity and effective reduction and capping by bacterial biomolecules. In contrast, other ratios resulted in blue-shifted and broadened peaks, typically associated with the presence of impurities, insufficient reducing agents, leading to NPs aggregation. To evaluate the effect of pH on SeNPs synthesis, pH values ranging from 5 to 9 were tested (Fig. 2D). Visible red color, indicating SeNPs formation, was observed at pH 7 and 8, suggesting optimal conditions for stable NPs production. however, at pH 9, a blue shift in the absorbance spectrum was detected without the characteristic red color, indicating the formation of unstable NPs. These findings highlight pH 7–8 as the most suitable condition for producing stable and well-dispersed SeNPs, in accordance with Ali et al. [44].

### Characterization of SeNPs and Se/CS NC

**Fig. 3:**
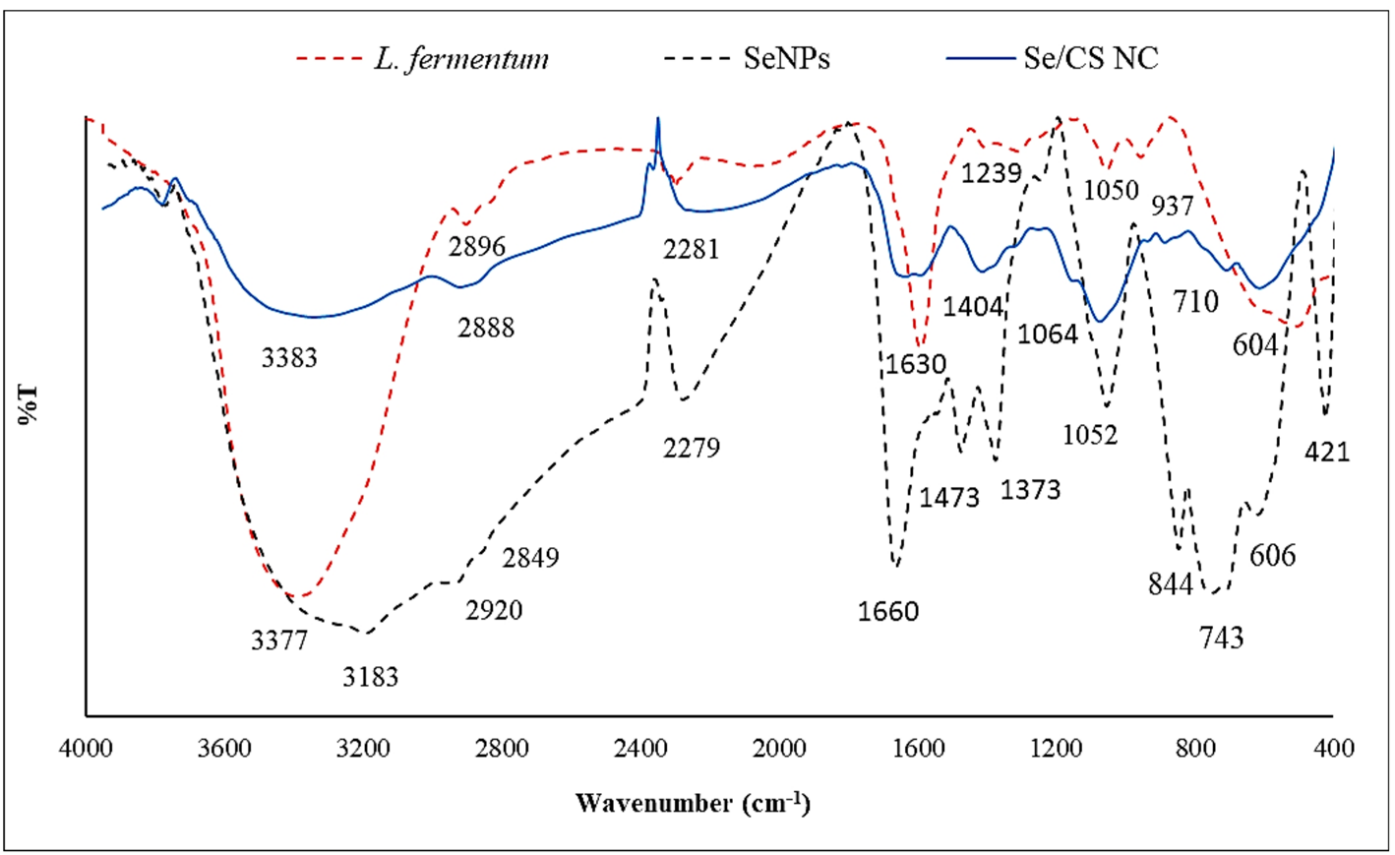
FT-IR spectra of the *L. fermentum* cell-free supernatant, biosynthesized SeNPs and Se/CS NC

Nanocomposite was successfully prepared by coupling SeNPs with CS, as confirmed by various analyses including FT-IR, XRD, TEM, and Zeta potential measurements. A FT-IR spectrometer was used to detect and identify the presence of functional groups involved in the synthesis of SeNPs in the range of 400–4000 cm^− 1^ (Fig. 3). The FT-IR spectrum of the *L. fermentum* cell-free supernatant alone was generated and showcases prominent peaks corresponding to various functional groups, such as O–H (molecular water) stretching at 3377 cm^− 1^, C=O stretching (amides/carboxylic acids) at 1660 cm^− 1^, 1239 cm^− 1^, and 1373 cm^− 1^, and C–H stretching (aliphatic compounds) at 2896 cm^− 1^ originating from the bacterial metabolites. These specific functional groups present in the *L. fermentum* cell-free supernatant including proteins, carbohydrates, ad organic acids are evident in the control spectrum that were shifted and changed in peak positions, changes in intensity, or disappearance of certain peaks in the SeNP spectrum (relative to the *L. fermentum* control) confirm their involvement in both the reduction of selenite ions and the stabilization/capping of the nascent SeNPs. For instance, changes in the amide I and II bands or hydroxyl stretching bands provide strong evidence for the role of proteins and polysaccharides, respectively, in the synthesis process.

The FT-IR spectrum of the SeNPs exhibits a significant peak at 3183 cm^−1^, which might be attributed to the O–H stretching in molecular water or the N–H stretching in proteins [45]. The peak at 1660 cm^−1^ was attributed to the bending vibration of the amide I peak from proteins, while the peak at 1239 cm^−1^ corresponded to the presence of the amide III group [41]. The bands observed at 2920 and 2849 cm^−1^ represented the stretching vibrations of aliphatic CH_2_ groups [46].

The band observed at 1373 cm^−1^ is specifically assigned to the symmetric stretching vibration (*ν*_s_​, corresponding to the C−O character) of the carboxylate anion (COO^−^). This indicates that amino acid residues and organic acid metabolites, which contain these functional groups, are actively involved in the stabilization and capping of the synthesized SeNPs. This peak, paired with the shifts observed in the amide I/II region, collectively confirms the critical role of protein and organic acid components from the *L. fermentum* metabolites in the green synthesis process [47].

The band at 1373 cm^−1^ was assigned to symmetric stretching of carboxylate groups (COO⁻), suggesting the involvement of amino acid or protein residues in the stabilization of NPs [48]. The presence of C–O was confirmed by stretching vibrations observed at 1052 cm^−1^ [49]. The peaks at 421, 606, 743, and 844 cm^−1^ serve as strong evidence for the successful formation of SeNPs. The spectrum of the Se/CS NC showed noticeable changes, indicating interaction between CS and SeNPs. A clear decrease in the intensity of the stretching bands of O–H and N–H at 3383 cm^−1^, C–H at 2888 cm^−1^, amide I at 1630 cm^−1^, COO^−^ at 1404 cm^−1^, and C–O at 1052 cm^−1^ was observed, suggesting the involvement of hydroxyl, carboxylate and amine groups from CS in the stabilization or binding with SeNPs [50]. Furthermore, the decrease in the intensity of the peak at 606 and 743 cm^−1^, associated with Se–O bending vibrations, further supports the successful integration of CS with SeNPs [51].

**Fig. 4:**
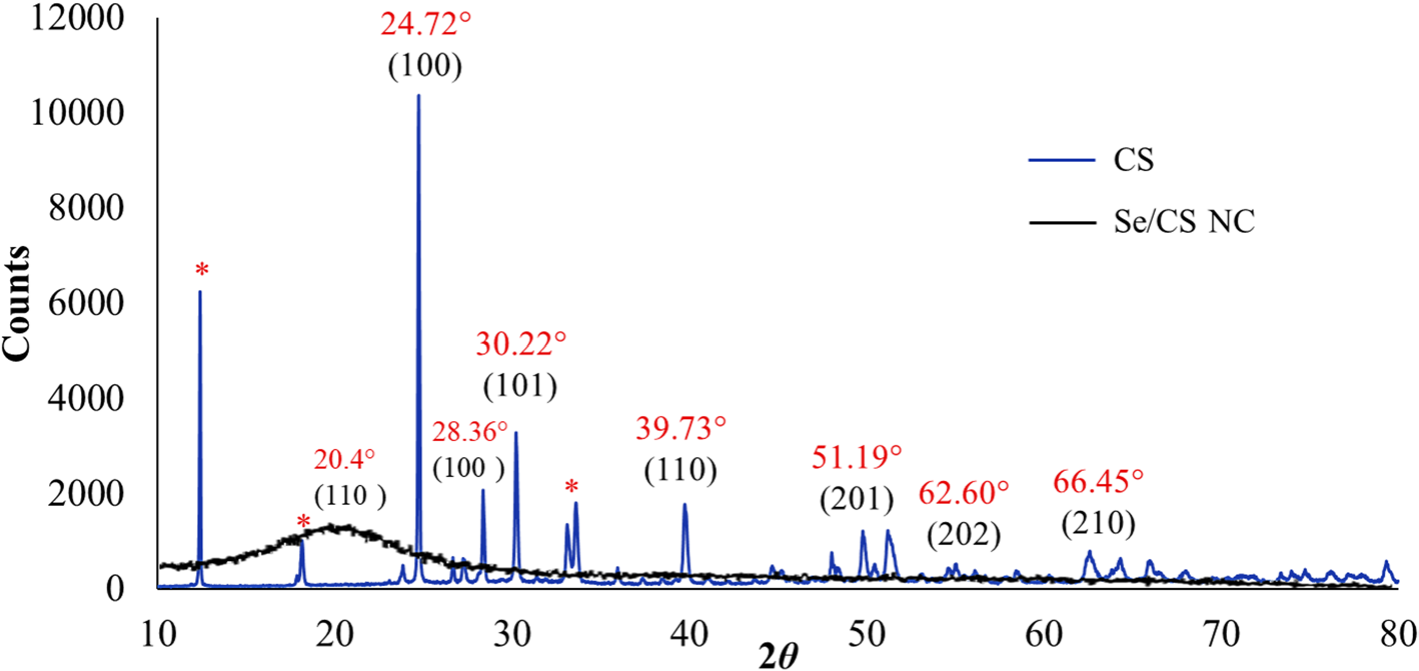
XRD spectrum of CS and Se/CS NC

The XRD analysis offered additional information on the structural characteristics of the nanomaterials. The pure CS pattern (black line, Fig. 4) clearly exhibits the characteristic broad, semi-crystalline peak centered around 20.4°, which is consistent with the previous literatures [52, 53]. A strong, sharp peak appears in the range of 19° to 21°. This peak is the most characteristic feature of CS’s crystalline structure and is often attributed to the (110) crystal planes of the CS unit cell (Form II crystal structure). The high degree of deacetylation (≥ 85%) promotes greater regularity in the polymer chains, which allows for stronger inter- and intra-molecular hydrogen bonding and, consequently, a more defined crystalline peak. Upon the incorporation of SeNPs, the resulting Se/CS nanocomposite (blue line) shows significant changes in this region. The major characteristic peak of CS is broadened and shifted to a lower angle (approximately 20.1°). This shift, combined with a noticeable decrease in the peak’s intensity, suggests a reduction in the overall crystallinity of the CS matrix. This phenomenon is likely due to the disruptive intercalation of the SeNPs and the resulting strong intermolecular interactions (such as hydrogen bonding) occurring between the Se particles and the polymer chains. These interactions hinder the organized packing of the CS chains, leading to a less ordered, more amorphous structure in the nanocomposite [54]. The observed diffraction peaks at 2*θ* angles of 24.72°, 28.36°, 30.22°, 39.73°, 51.19°, 62.60°, and 66.45° matched the Bragg reflections from the (100), (100), (101), (110), (201), (202) and (210) crystallographic planes, respectively (blue line, Fig. 4). The intense peak at 24.72° suggests a high degree of crystallinity. The diffraction peaks matched well with the standard hexagonal phase of Se, as reported in JCPDS no. 06-0362. Our results agree well with El-Saadony et al. and Mohamed & El-Zahed [24, 55]. Debye-Scherrer equation (D = kλ/βcos*θ*; where D; average crystalline particle size, λ; wavelength of x-ray (1.5406 Å), k; Scherer’s constant (0.9), *θ*; diffraction angle, and β; XRD peak full width at half maximum) was applied to calculate SeNPs size. The calculated average size was 38.19 nm.

**Fig. 5:**
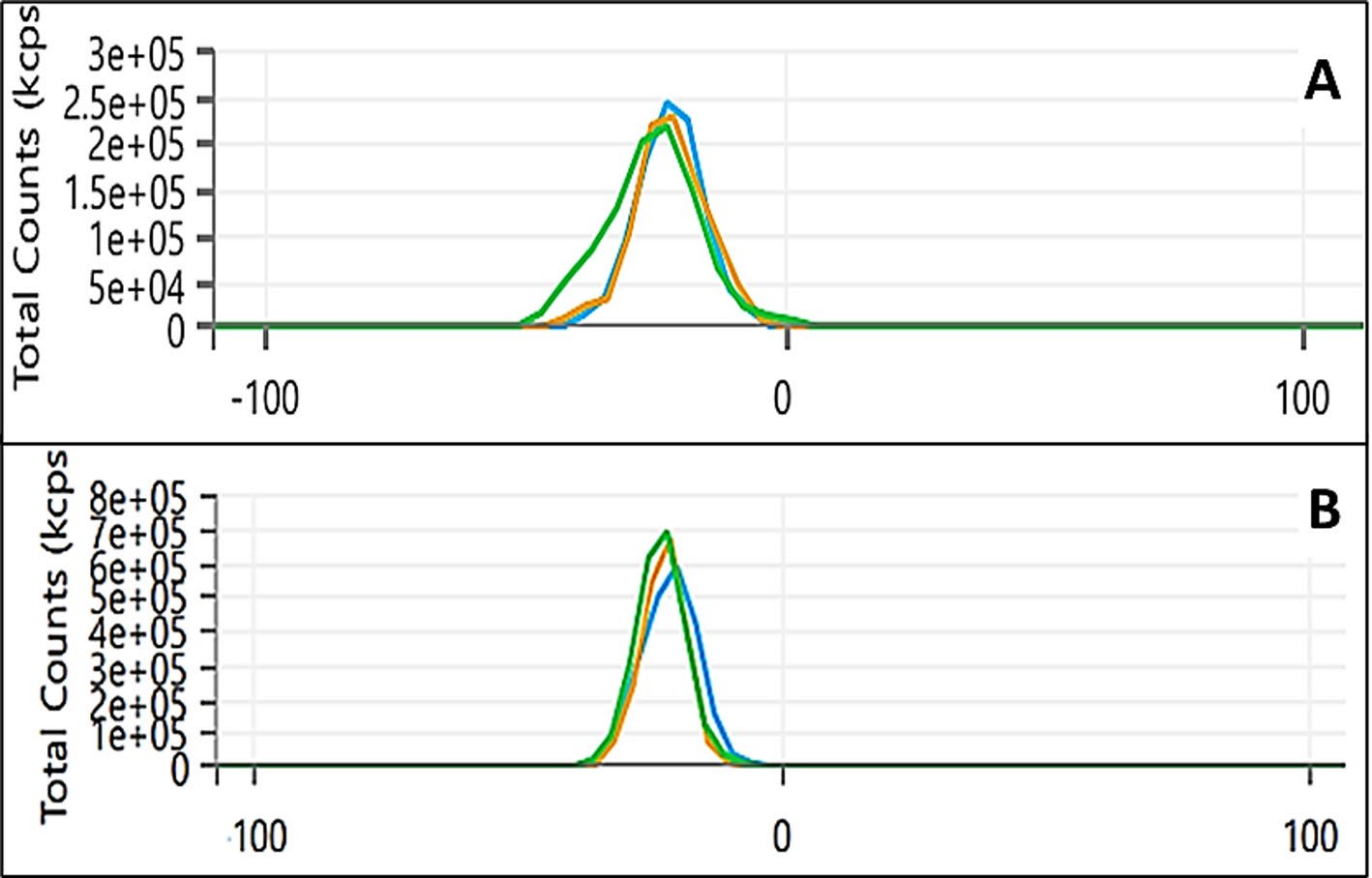
Zeta potential analysis of SeNPs (**A**), and Se/CS NC (**B**)

Zeta potential serves as an important parameter in evaluating the stability of NPs’ colloidal dispersions. NPs with a larger Zeta potential show greater stability due to the stronger electrostatic repulsion between them [56]. Zeta potential results showed that the biosynthesized SeNPs of *L. fermentum* had a negative charge of − 30.21 ± 3.5 mV, while Se/CS NC had a negative charge of − 21.84 ± 4.7 mV, which results in a force of repulsion between the NPs and indicates their high stability (Fig. 5). The obtained results matched with *Bacillus* sp. SeNPs as reported by Ullah et al. [41]. Additionally, Laslo et al. [57] reported a negative charge of – 23 mV for SeNPs biosynthesized by *L. casei*. Moreover, the negative surface charge has been associated with notable antiviral activity by facilitating electrostatic interactions with positively charged viral components, potentially disrupting viral attachment or entry processes [58, 59].

The negative Zeta potential of the final Se/CS NC (− 21.84 mV) is indeed unexpected given the traditionally positive charge of free CS (due to its protonated amine groups, NH_3_^+^​). This result was attributed to two main factors [60]. First, the initial negative charge of the biosynthesized SeNPs (− 30.21 mV) is very strong. The SeNPs are synthesized and stabilized by various anionic components (e.g., proteins, polysaccharides, and other biomolecules) inherited from the *L. fermentum* supernatant. Secondly, when the positively charged CS is added, it interacts with the strongly negative surface of the SeNPs via electrostatic forces. In this interaction, the CS may partially wrap the NPs, but the overall surface potential of the final complex is still dictated by the unshielded negative charge of the SeNPs surface, or the CS-amine groups are complexed or shielded by the strong anionic species surrounding the SeNPs, leading to a residual negative potential for the overall nanocomposite. The measured value of − 21.84 mV is less negative than the free SeNPs (− 30.21 mV), confirming that the positive CS has partially neutralized the overall surface charge, but not enough to flip it to a positive value. This final negative value is still well outside the critical range (typically ± 20 mV), confirming the high stability of the synthesized nanocomposite due to electrostatic repulsion.

**Fig. 6:**
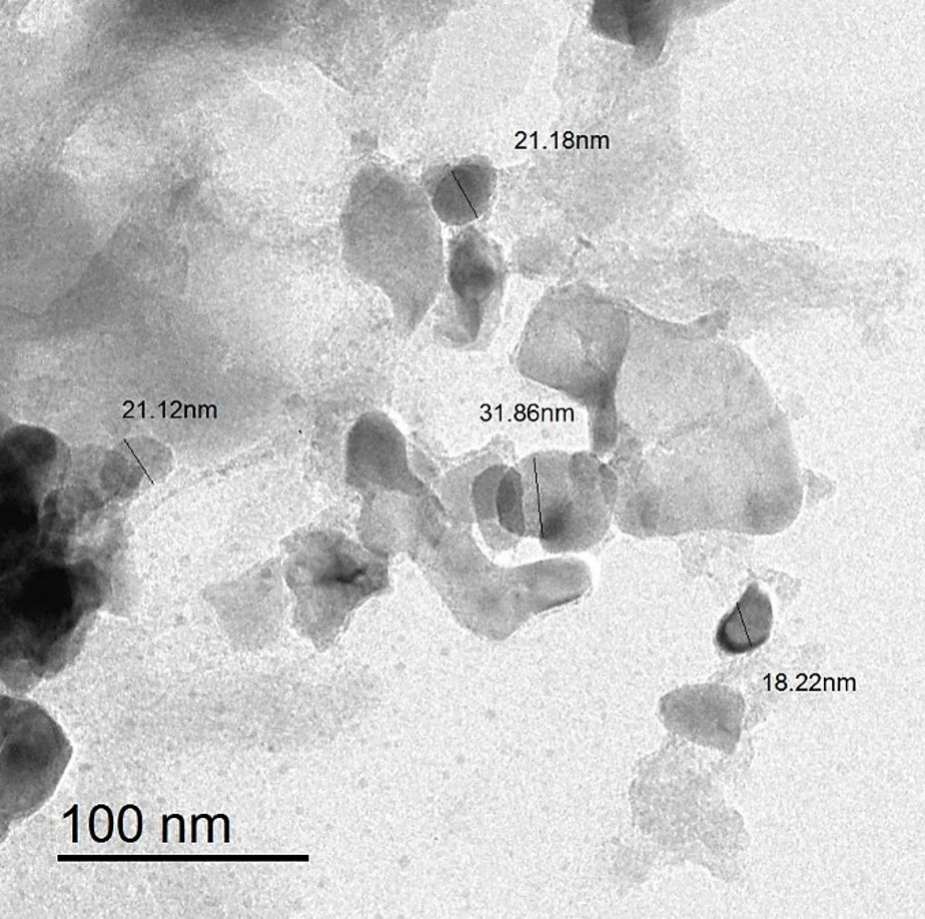
TEM of Se/CS NC. Bars scale = 100 nm

TEM was used to study the morphology of *L. fermentum*-SeNPs. The TEM micrograph revealed that the produced NPs were spherical-like in shape, with an average diameter between 18 and 32 nm (Fig. 6). The current results (18–32 nm) are consistent with those of Mohamed and El-Zahed [24] who biosynthesized SeNPs with a size range of 17–30 nm, which matched the data calculated from XRD. For instance, the size of SeNPs produced by *L. lactis* was reported to be 38–152 nm, and those produced by *L. casei* ranged from 150 to 400 nm [61, 62]. The size of NPs plays an important role in their application, as smaller NPs are believed to be more effective in biological activities.

### Cytotoxicity assay

**Fig. 7:**
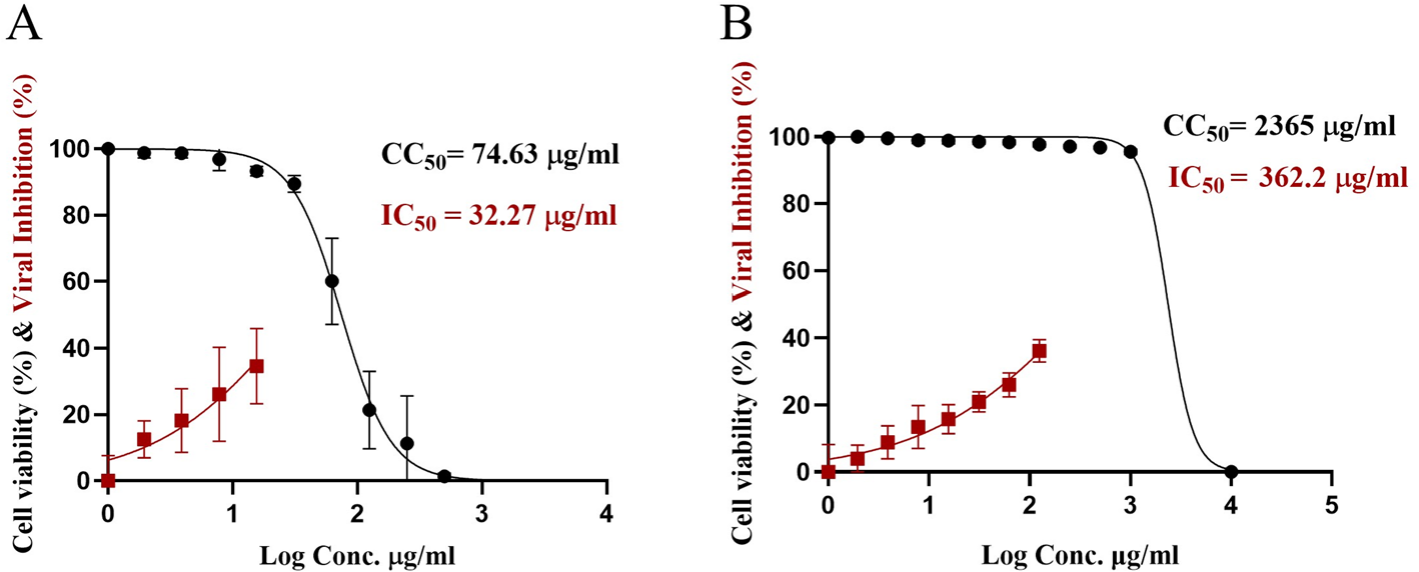
Cytotoxic concentration 50 (CC_50_) and inhibitory concentration 50 (IC_50_) of the biosynthesized SeNPs (**A**) and SE/CS NC (**B**) against SARS-CoV-2

The cytotoxicity of a product on human cell lines must be studied to assess its safety [17, 63]. Generally, SeNPs provide environmentally safe, low-toxicity, and biologically active compounds [64]. This study examined the cytotoxicity of biosynthesized SeNPs and Se/CS NC against the Vero E6 cells (Fig. 7). Unmodified SeNPs demonstrated cell viability sharply declining at concentrations above 125 µg/ml and a calculated CC_50_ of approximately 74.63 µg/ml. However, at lower concentrations (≤ 31.25 µg/ml), cell viability remained consistently above 90%. In contrast, Se/CS NC exhibited markedly improved cytocompatibility, maintaining over 96% cell viability across all tested concentrations up to 1000 µg/ml, with a CC_50_ of 2365 µg/ml. These data show that chitosan functionalization considerably reduces SeNPs cytotoxicity. Chitosan’s steric and electrostatic characteristics likely promote colloidal stability and decrease toxicity [65]. The mechanism of cytotoxicity was previously explained as the ion channels carry SeNPs to the cell membrane, interacting with DNA nitrogen bases or intracellular proteins to cause cell cycle arrest, mitochondrial malfunction, DNA breakage, and apoptosis [66, 67]. Generally, a chemical is non-cytotoxic if its CC_50_ value exceeds 90 µg/ml [68]. This reveals that SeNPs have a narrow therapeutic window compared to their Se/CS NC derivative, and the biosynthesized Se/CS NC is safe for human cell types.

### Antiviral activity of SeNPs and Se/CS NC

As antimicrobials, nanoparticles can kill bacteria, fungi, algae, archaea, and viruses. Inhibiting cell membrane formation, energy transmission, harmful reactive oxygen species (ROS) production, and RNA and DNA synthesis are their modes of action. A variety of nanomaterials have been used as antiviral agents to combat viral infections [69]. The application of nano-based therapy to target viruses, including SARS-CoV-2, has been effective in previous research studies [70–73]. In this study, antiviral assays revealed that both SeNPs and Se/CS NC inhibited SARS-CoV-2 in a concentration-dependent manner. SeNPs exhibited an IC_50_ of 32.27 µg/ml, but their antiviral efficacy rapidly diminished at lower concentrations, with only 12.26% inhibition observed at 1.95 µg/ml and no detectable effect at 1 µg/ml (Fig. 7A). In comparison, Se/CS NC demonstrated robust antiviral activity across a broader concentration range, with an IC_50_ of 362.2 µg/ml (Fig. 7B). While Se/CS NC required a higher concentration than SeNPs to inhibit 50% of viral activity, its substantially reduced cytotoxicity translated into a much improved selectivity index (SI = 6.53 for Se/CS NC compared to 2.3 for SeNPs). This enhanced balance between antiviral effectiveness and cell safety positions Se/CS NC as a desirable candidate for advancing antiviral therapy options. Chitosan nanoparticles (CNPs) are an effective and safe drug carrier that improves therapeutic efficacy and reduces side effects in antiviral applications [15, 74]. CNPs are known to be biocompatible, biodegradable, non-immunogenic, and nontoxic [15].

Se/CS NC offers a more favorable safety profile than other antiviral nanomaterials, such as AgNPs, ZnO NPs, and CuO NPs. Se/CS NCs demonstrate significantly reduced cytotoxicity towards mammalian cells, owing to the biocompatible characteristics of chitosan and the regulated release behavior of SeNPs [75, 76]. In contrast to AgNPs, which, despite their significant antiviral effectiveness, are associated with oxidative stress, apoptosis, and tissue damage at therapeutic concentrations [77–79], Se/CS NCs present a safer option, even exhibiting the capacity to alleviate oxidative and histological damage caused by AgNPs in vivo [75]. Likewise, although ZnO nanoparticles have a superior selectivity index compared to AgNPs, their cytotoxicity is still greater than that of Se/CS NC, especially at increased concentrations [77, 80].

### Mechanism of antiviral action

**Fig. 8:**
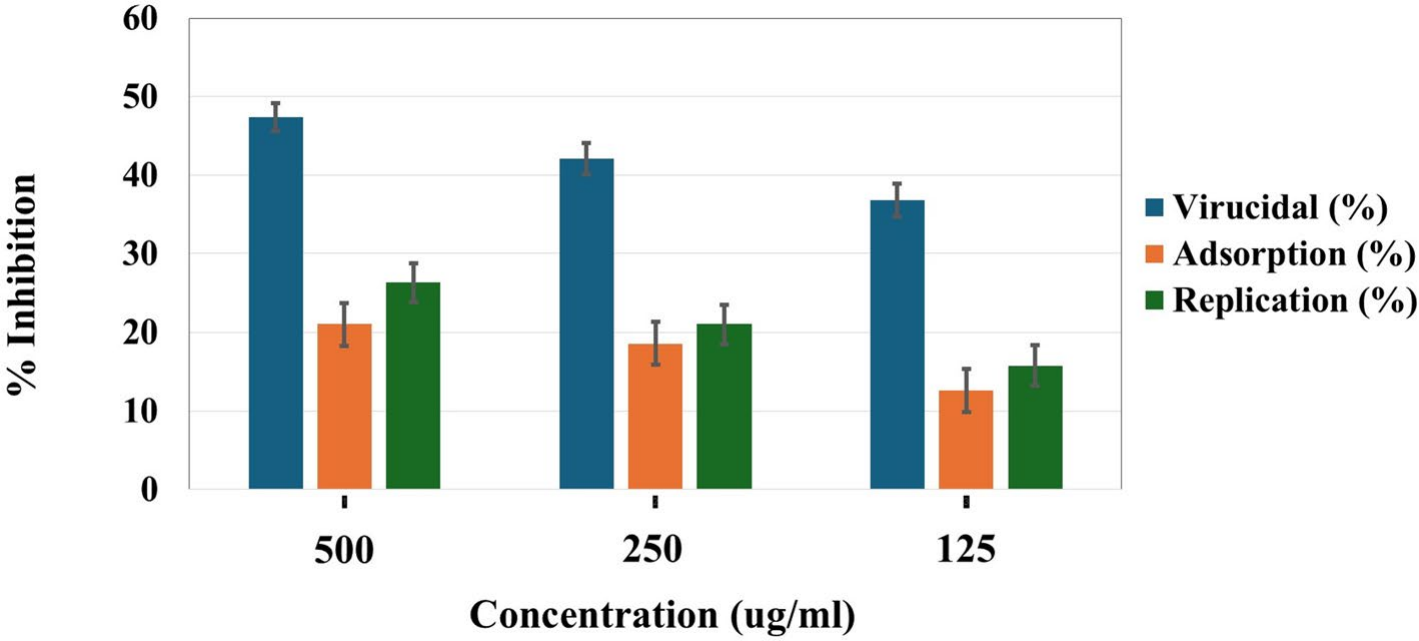
Antiviral mode of action for the biosynthesized Se/CS NC with different concentrations against SARS-CoV-2

Since Se/CS NC had a better safety profile than SeNPs, it was selected further to explore its antiviral mode of action against SARS-CoV-2. Se/CS NC was assessed at three stages of the viral replication cycle: direct virucidal activity, adsorption inhibition, and viral replication inhibition to further elucidate its mechanism of action. At a concentration of 500 µg/ml, Se/CS NC achieved a 47.4% reduction in plaque formation during direct virus exposure, indicating a strong virucidal effect. Se/CS NC blocked viral adsorption and replication at rates of 21.0% and 26.3%, respectively (Table 1; Fig. 8). Our findings show that Se/CS NC can directly inactivate viral particles, making it an efficient virucidal agent, likely due to interactions with the viral envelope or spike protein. This mechanism is consistent with previous reports that SeNPs have 87.5% virucidal activity against SARS-CoV-2 [81]and that combining antiviral agents such as nanoparticles and chitosan may have a more substantial synergistic effect [82].

**Table 1:** The mode of antiviral action of Se/CS NC

Mode of action	Se/CS NC Conc. µg/ml	Virus control pre-treatment (PFU/ml)	Viral count post-treatment (PFU/ml)	% Viral inhibition
Virucidal	500	1.9 * 10^6^	(1.00 ± 0.03) * 10^6^	47.4 ± 1.79
	250		(1.10 ± 0.04) * 10^6^	42.1 ± 2.01
	125		(1.20 ± 0.04) * 10^6^	36.8 ± 2.15
Viral adsorption	500	1.9 * 10^6^	(1.50 ± 0.05) * 10^6^	21.0 ± 2.71
	250		(1.55 ± 0.05) * 10^6^	18.4 ± 2.73
	125		(1.66 ± 0.06) * 10^6^	12.6 ± 2.76
Viral replication	500	1.9 * 10^6^	(1.40 ± 0.05) * 10^6^	26.3 ± 2.51
	250		(1.50 ± 0.05) * 10^6^	21.0 ± 2.45
	125		(1.60 ± 0.06) * 10^6^	15.8 ± 2.55

Literature supports the broader antiviral potential of chitosan-based nanomaterials well. For instance, silymarin-loaded chitosan nanoparticles have demonstrated potent antiviral activity against SARS-CoV-2, with IC₅₀ values as low as 0.8 µg/ml, while maintaining excellent biocompatibility [13]. Additionally, treating H3N2 infections with SeNPs and chitosan lowered toxicity and inhibited viral multiplication, cell disintegration, and aggregation. It also stabilized mitochondrial membrane potential and decreased ROS generation. It also reduced H3N2-induced inflammation, late apoptosis, and infection [83].

Even though the preliminary in vitro results have been encouraging, comprehensive in vivo validation is still required for Se/CS NC. To better understand the route of action, mechanistic investigations should investigate the interactions that Se/CS NC has with the proteins of the viral or host cell. In addition, the mucoadhesive characteristics of chitosan could be utilized to generate inhalable or intranasal formulations for targeted distribution to the respiratory system [84, 85].

All things considered, chitosan-functionalized selenium nanoparticles are a promising antiviral formulation against SARS-CoV-2. They have vigorous virucidal activity, significantly better cytocompatibility than unmodified SeNPs, and a good safety record that encourages additional therapeutic development and nanocoating formulation.

## Conclusions

*L. fermentum* biosynthesized SeNPs, which were optimized and combined with CS to evaluate their antiviral action against COVID-19. The optimum conditions for SeNPs production indicated that the production rates increased by increasing the concentration of Na_2_SeO_3_ to 30 mM, and the temperature until 40 °C. The optimization conditions referred to a mixing ratio between *L. fermentum* cell-free supernatant and Na_2_SeO_3_ by a 1:1 volume/ratio and pH 7–8 as the most suitable condition for producing stable and well-dispersed SeNPs. The prepared nanocomposite appeared as spherical-like particles, which displayed high stability due to the presence of proteins and their highly negative surface charge. SeNPs alone cause dose-dependent cytotoxicity in SARS-CoV-2-infected Vero E6 cells, reducing cell viability above 125 µg/ml. At dosages ≥ 250 µg/ml, Se/CS NC demonstrated > 96% cell viability and 95% virus replication suppression. Research indicates that virucidal activity is the primary antiviral mechanism, having a 47.4% inhibition at 500 µg/ml. The future research plan will focus on a systematic optimization study using DLS to optimize critical parameters, including the concentration of the SeO_3_^2−​^ precursor, the ratio of the *L. fermentum* supernatant to the precursor solution, and the optimal incubation temperature, following established methods in nanobiotechnology. This planned inclusion of DLS in our follow-up research will undoubtedly enhance the scalability and industrial relevance of our green synthesis protocol. In addition, the use of field emission scanning electron microscopy (FE-SEM) could help obtain ultra-high-magnification images to confirm the spherical morphology and assess the uniformity and smoothness of the surface of both the bare SeNPs and the final Se/CS NC.

## Data Availability

All data supporting the findings of this study are available within the paper.
